# Health-related factors predict return to custody in a large cohort of ex-prisoners: new approaches to predicting re-incarceration

**DOI:** 10.1186/s40352-015-0022-6

**Published:** 2015-05-20

**Authors:** Emma G Thomas, Matthew J Spittal, Faye S Taxman, Stuart A Kinner

**Affiliations:** 1 Melbourne School of Population and Global Health, University of Melbourne, 207 Bouverie Street, Parkville, Victoria, 3010 Australia; 2 Criminology, Law & Society, College of Humanities and Social Sciences, George Mason University, 4087 University Drive, Fairfax, Virginia, 22030 USA; 3 School of Medicine, University of Queensland, 288 Herston Road, Herston, Queensland, 4006 Australia; 4 Griffith Criminology Institute, Griffith University, 176 Messines Ridge Rd, Mt Gravatt, Queensland, 4121 Australia

**Keywords:** Prisoner health, Ex-prisoner health, Recidivism, Re-incarceration, Survival analysis

## Abstract

**Background:**

Numerous poor health outcomes have been documented in the world’s large and growing population of prisoners and ex-prisoners. Repeat justice involvement and incarceration is normative for ex-prisoners in most countries. This study aimed to identify important health-related predictors of re-incarceration and to quantify their contribution to predicting re-incarceration.

**Methods:**

Participants were 1 325 adult ex-prisoners in Queensland, Australia. We developed a multivariate Cox proportional hazards model for re-incarceration including health-related covariates from a pre-release survey.

**Results:**

In addition to well-established risk factors (criminal history, drug-related sentence, younger age, male gender and Indigenous ethnicity), several health-related variables were important risk factors for re-incarceration in multivariate analyses, including risky use of cannabis (hazard ratio 1.27; 95% confidence interval 1.06, 1.51), amphetamines (HR 1.20; 95%CI 0.99, 1.46) or opioids (HR 1.33; 95%CI 1.08, 1.63) prior to incarceration, central nervous system medication prescription (HR 1.28; 95%CI 1.06, 1.54), reporting that maintaining physical health post-release was not important (HR 1.52; 95%CI 0.98, 2.36) and poverty prior to incarceration (HR 1.24; 95%CI 1.02, 1.52). Sedentary behaviour (HR 0.82; 95%CI 0.68, 1.00), obesity (HR 0.81; 95%CI 0.64, 1.02), multiple lifetime chronic illnesses (HR 0.85; 95%CI 0.71, 1.01) and a history of self-harm (HR 0.72; 95%CI 0.59, 0.88) were associated with a reduced risk of re-incarceration. Inclusion of health-related variables in the model improved prediction of re-incarceration compared to a model with only demographic and criminal justice predictors, leading to an increase in adjusted proportion of explained variation of 0.051 (95%CI 0.031, 0.107).

**Conclusions:**

Health-related factors predict re-incarceration after adjustment for demographic and criminal justice factors. Further research is required to establish the reproducibility of our findings and understand the causal pathways linking health at release from prison to re-incarceration.

**Electronic supplementary material:**

The online version of this article (doi:10.1186/s40352-015-0022-6) contains supplementary material, which is available to authorized users.

## Background

The global prison population is large and growing, with in excess of 11 million adults in custody on any day (Walmsley 2013) and an estimated 30 million people released from custody annually (UNODC 2008). In many countries, recidivism is normative: for example, in the United States 55% of ex-prisoners are re-incarcerated within five years of release (Durose et al. 2014), and in Australia, 40% are re-incarcerated within two years (SCRGSP 2014). Repeat incarceration exerts a significant burden on individuals, families and society as a whole. Incarceration is associated with deteriorating health (Massoglia 2008a, 2008b; Brinkley-Rubinstein 2013), increased risk of future offending (Durose et al. 2014) and worsening socioeconomic outcomes for offenders and their families, including homelessness (Dyb 2009) and reduced income (Western et al. 2001).

A growing body of literature has documented poor health outcomes in offending populations both in custody (AIHW 2013) and after return to the community (Kinner 2006; Cutcher et al. 2014). Very high rates of substance dependence (Fazel et al. 2006) and mental illness (Fazel and Danesh 2002) have been observed among prisoners and ex-prisoners. Both groups are at greatly increased risk of communicable disease, including sexually transmitted infections (STIs), hepatitis B and C, and human immunodeficiency virus (HIV) (Fazel and Baillargeon 2011; Butler et al. 2013). The prevalence of non-communicable diseases is also elevated, although this issue is under-recognised (Herbert et al. 2012; Binswanger et al. 2009). Following release, ex-prisoners are at increased risk of mortality associated with suicide (Pratt et al. 2006), drug overdose (Merrall et al. 2010) and injury (van Dooren et al. 2013), as well as death from natural causes (Spaulding et al. 2011). Despite this, in most countries the health needs of prisoners receive relatively little policy attention (Lines 2006) and remarkably little is known about broader health outcomes after release from prison, or how these may shape future offending trajectories.

There has been extensive research on risk factors for criminal recidivism. However, these studies have typically relied on routinely collected data, limiting the pool of potential predictors available for study and necessitating crude variable measurement. Health-related variables have rarely been investigated, and many studies have focussed on developing risk assessment tools based only on criminal justice factors, such as number of prior arrests, incarcerations or infractions in prison (Singh and Fazel 2010). Two relatively dated meta-analyses of this literature have identified key risk factors for recidivism in adult offenders, including a history of criminal activity, juvenile delinquency, criminogenic needs, antisocial personality and socialisation with other prisoners (Bonta et al. 1998; Gendreau et al. 1996). Extensive research has also shown that drug and alcohol abuse (Dowden and Brown 2002) and mental disorder (Baillargeon et al. 2010), including psychotic (Fazel and Yu 2011) and personality disorders (Yu et al. 2012), are predictors of recidivism*,* although the mechanisms underpinning these associations remain unclear, and there is evidence that the association between mental disorder and offending may be mediated by factors such as substance use (Rezansoff et al. 2013).

There is currently a paucity of research on other health-related predictors of recidivism and many existing studies have been cross-sectional in design, such that it is difficult to disentangle possible causal relationships. Previous prospective studies have typically employed simple, binary measures of recidivism that fail to characterise the amount of time that individuals spend in the community before reoffending or returning to custody. At least two recent studies in the United States have investigated the link between recidivism and other health and social factors (Freudenberg et al. 2005; Fu et al. 2013). In a study of adult women and adolescent males, Freudenberg et al. (2005) found that having health insurance after release greatly reduced the odds of re-arrest up to 15 months later. For males, employment post-release reduced the risk of re-arrest, and for females, homelessness increased this risk. However, the authors found no evidence for an association between recidivism and self-reported physical health problems, post-release social support or health service participation. Fu et al. (2013) found that, among HIV positive adults released from prison, having health insurance or medical benefits in the first 30 days following release greatly reduced the odds of any re-incarceration up to six months after release, while homelessness prior to index incarceration increased the risk of return to custody. They found no evidence for a link between recidivism and educational attainment or employment prior to index incarceration.

In the present study, we employ time-to-event analyses to investigate associations between re-incarceration and a wide range of health indicators measured prior to release. Our focus on health-related variables is important for two key reasons. First, these characteristics are often dynamic and open to change; health-related factors that prospectively predict re-incarceration thus represent potential points of intervention for breaking the cycle of release and return to custody. Second, improved prediction of recidivism using health-related characteristics that are easily measured prior to release could enable better identification of high-risk individuals and hence aid in targeting transitional interventions. Any identified associations between health and criminal recidivism may also have implications for post-release healthcare delivery in countries such as Australia, where there is universal access to affordable or free primary healthcare and hospitals (AIHW 2012), but where ex-prisoners are at much greater risk of hospitalisation than the general population (Alan et al. 2011). In the United States, such questions are increasingly relevant as healthcare reform dramatically improves the potential for health insurance coverage among former inmates (Cuellar and Cheema 2012).

Using data from a large cohort of adult prisoners and ex-prisoners in Queensland, Australia, the aims of this study were to (1) identify health-related predictors of re-incarceration, and (2) examine whether the addition of health-related factors improves prediction of re-incarceration in a model that already includes criminal justice and demographic variables.

## Methods

### Setting

Data for this study came from a randomised controlled trial (RCT) of a re-entry intervention for adult ex-prisoners in Queensland, Australia (Kinner et al. 2013; Kinner et al. 2014). Baseline interviews were conducted within six weeks of expected release from custody, and before randomisation. Baseline data were collected using a structured paper questionnaire in confidential interviews typically lasting 60–90 minutes. Participants in the intervention group received a personalised booklet summarising their health status and identifying appropriate community health services. Trained workers made weekly telephone contact in the first four weeks after release from prison to identify health needs and facilitate health service utilisation. Participants in the control group received a letter summarising their health status at release and usual care post-release. Follow-up interviews with all participants were conducted approximately one, three and six months post-release. The primary outcome of the RCT was health care utilisation at six months post-release. Here we focus on the baseline data because (a) this reflects the information that is available when a prisoner is released into the community, and can thus be used to predict future incarceration, and (b) considering prospective associations with re-incarceration allows us to identify potential targets for preventive intervention.

### Participants

Participants were sentenced adult prisoners from seven Queensland prisons, recruited August 2008 – July 2010. Eligible participants were within six weeks of expected release (full-time or parole) and able to provide informed, written consent. Remand (pre-trial) prisoners were excluded due to uncertainty regarding release. Women were oversampled to ensure sufficient numbers for sex-stratified analyses.

Recruitment occurred via consecutive sampling and the recruitment fraction was 80% (Kinner et al. 2014). Potential participants were identified from prison records and were seen by trained interviewers in a private location. Interviewers explained the study and provided an information sheet in plain language. Eligible participants were invited to sign a consent form. Participants received AU$10 remuneration, transferred into their prison trust account.

### Measures

#### Baseline variables included in models for return to custody

As this study was a re-analysis of existing data, when selecting covariates for inclusion in our models for re-incarceration we were limited by the questions posed in the baseline survey, although this included a broad range of variables. We selected key criminal justice and demographic predictors based on existing evidence. The World Health Organization defines health as “a state of complete physical, mental and social well-being and not merely the absence of disease or infirmity” (WHO 2006). We aimed to include a range of health indicators that was broadly consistent with this definition and captured the most prevalent morbidities in our cohort. We also included variables indicating health involvement, which we defined as the motivation and capacity of an individual to manage his/her own health. In addition, we included indicators of socioeconomic status because these are often correlated with both health and return to custody, and thus represent potential confounders. As important social determinants of health (Marmot and Wilkinson 2005), these variables are also of interest in their own right. We thus categorised the predictors examined according to six domains: substance use, mental health, physical health, social support, health involvement and socioeconomic factors. Where possible, we chose at least one objective and one subjective indicator within each domain. We constructed variables from previously validated instruments when available.

Table 1 describes the precise definition and measurement of baseline variables and shows the number of participants with missing data on each variable. Demographic descriptors included age, sex and Indigenous status. Criminal justice variables included historical incarcerations (adult or juvenile), length of sentence, drug-related sentence and income from illegal activities prior to incarceration.

**Table 1:** **Description of potential predictors of time to re-incarceration and number of participants with missing data (N=1325)**

**Variable**	**Description** ^**1**^ ***(Number with missing data out of full sample)***
*Demographic*	
Age	Age at release, categorised into three groups: 18 to 24 years, 25 to 39 years, and 40 years and above *(0)*
Female	Female gender *(0)*
Indigenous	Australian Aboriginal and/or Torres Strait Islander *(0)*
*Criminal justice*	
Prior adult incarceration	Any prior incarcerations aged ≥17 years *(2)*
Juvenile incarceration	Any incarcerations aged <17 years *(13)*
Any income from illegal activities	Any income from illegal activities in the four weeks before incarceration *(2)*
Longer sentence (≥6 months)	QCS records *(0)*
Drug-related sentence	Drug related sentence according to QCS records *(13)*
*Substance use*	
High risk drinking	Scored ≥16 on the Alcohol Use Disorders Identification Test (Babor et al. 2001), indicating high risk drinking to possible alcohol dependence, with reference to drinking in the year before prison *(11)*
Risky cannabis use	Scored ≥4 on the cannabis section of the Alcohol, Smoking and Substance Involvement Screening Test (ASSIST) (Humeniuk et al. 2010), indicating moderate to high risk use, with reference to drug use in the three months before prison *(1)*
Risky amphetamine use	Scored ≥4 on the amphetamine section of the ASSIST, indicating moderate to high risk use, with reference to drug use in the three months before prison *(2)*
Risky opioid use	Scored ≥4 on at least one of the heroin section or the other opiates section of the ASSIST, indicating moderate to high risk use, with reference to drug use in the three months before prison *(3)*
*Mental health*	
History of self-harm	Any history of self-harm, including attempted suicide *(0)*
CNS medications	Central Nervous System (CNS) medication (defined according to (MIMS 2014)) prescription at time of baseline interview, according to QCS health records accessed with participant consent *(94)*
Screens positive for intellectual disability	At least two of: scored <84.5 on the Hayes Ability Screening Index (HASI) (Hayes 2000); attended a special school; has been diagnosed with an intellectual disability *(22)*
High psychological distress	Scored ≥22 on the Kessler Psychological Distress Scale, indicating high to very high psychological distress (Kessler et al. 2002) *(5)*
*Physical health*	
Two or more chronic illnesses	Ever been diagnosed with at least two of: high blood pressure, high cholesterol, heart disease, diabetes, epilepsy or cancer/tumours *(0)*
Low physical health functioning	Scored ≤50.8 (the 25^th^ percentile of the sample) on the SF-36 Version 2 Physical Component Summary measure (Ware et al. 2000), Australian *T*-normed scores (ABS 1997) *(20)*
Obese	BMI > 30kg/m^2^, where BMI = weight/height^2^, weight and height from averages of two measurements taken by trained interviewers at baseline interview *(7)*
Sedentary	Fortnightly exercise participation <100 minutes *(4)*
Any STI	Ever been diagnosed with a sexually transmitted infection *(0)*
*Social support*	
Not married or de-facto	Not married or in a de-facto relationship at time of baseline interview *(0)*
No visits in past four weeks	Not visited in prison by any community contacts in the four weeks prior to baseline interview *(0)*
Low perceived social support	Scored ≤2 on at least two of five items and a total score of ≤18 (ENrICHD Investigators 2001) on the five item ENrICHD Social Support Inventory (Mitchell et al. 2003) *(4)*
Taken from family as child	Ever taken away from family as a child *(3)*
*Health involvement*	
Low patient activation	Scored ≤55.1 on the Patient Activation Measure, indicating lack of motivation to take an active role in own health care or lack of knowledge and confidence to do so (Hibbard et al. 2004) *(13)*
Physical health not important	Reports that it is not very or not at all important to maintain own physical health after release *(2)*
Mental health not important	Reports that it is not very or not at all important to maintain own mental health after release *(2)*
*Socioeconomic*	
Less than ten years schooling	Less than 10 years of school attended *(4)*
Below poverty line	Income in 4 weeks before incarceration below poverty line according to a published Australian standard (MIAESR 2014), accounting for dependents and marital status *(2)*
Unstable housing	No stable accommodation in the month prior to incarceration *(0)*
Unemployed	No part-time, full-time or casual employment in the 6 months prior to incarceration *(0)*

^**1**^Variables are from participant self-report unless otherwise stated.

We included substance use variables indicating risky use of the most common illicit drugs in our cohort (cannabis, amphetamines and opioids, including heroin) and heavy drinking as measured by validated screening tests (Babor et al. 2001; Humeniuk et al. 2010). Within mental health, we investigated historical self-harm or attempted suicide by self-report. Non-specific psychological distress was measured via a survey instrument (Kessler et al. 2002). Central Nervous System (CNS) medication prescription according to prison records was used as an objective proxy for mental illness. We constructed a compound indicator of possible intellectual disability using self-report and a screening test (Hayes 2002).

Within physical health, we constructed a variable indicating historical diagnoses of two or more chronic illnesses, since the proportion of participants reporting one or more illnesses was very high (82%). We measured physical health-related functioning, a broad indicator of physical wellbeing, using a validated survey instrument (Ware et al. 2000), and sedentary behaviour via self-report. Obesity was included as an objective, although imperfect, indicator of physical health. With regard to communicable diseases, we considered only STIs. Rates of HIV and lifetime hepatitis B infection were very low in our cohort (<1.5%), and while lifetime hepatitis C infection was comparatively common (30%), we excluded this variable because it was very strongly associated with illicit drug use (90% of those reporting a positive hepatitis C test also reported risky use of cannabis, amphetamines or opioids).

Indicators of social support included marital status, visits in prison (an objective measure), perceived social support using a validated survey instrument (Mitchell et al. 2003) and a survey item reporting separation from family as a child. We measured health involvement using a patient activation scale (Hibbard et al. 2004) and two survey items indicating the importance to the participant of maintaining his/her physical or mental health after release. We included indicators of four key socioeconomic factors that are often correlated with both health status and return to custody: education, income, housing and employment.

#### Time-to-event data

Release and re-incarceration dates were provided by Queensland Corrective Services (QCS) and covered the period from 1 January 2006 to 31 December 2013. Deaths among participants were identified for censoring purposes through probabilistic linkage with the National Death Index and covered the period from 1 January 2006 to 31 July 2013; any deaths between 1 August and 31 December 2013 would not have been identified, leading to possible under-ascertainment.

### Analyses

We performed time-to-event analyses using Cox proportional hazards regression. The outcome of interest was time to first re-incarceration. Participants were under observation upon release from index incarceration until return to custody, death or the study end date (31 December 2013).

We first performed univariate Cox proportional hazards regression using each candidate predictor of re-incarceration and then ran a full multivariate model including all predictors. Next, because of the large number of related variables in our models, we performed variable selection using the Least Absolute Shrinkage and Selection Operator (LASSO) (Tibshirani 1997) via the glmnet package in R (Friedman et al. 2010). The LASSO tends to select only one from among a group of highly correlated variables (Zou and Hastie 2005), thereby avoiding the potentially harmful effects of multicollinearity among covariates. We then ran a final reduced multivariate model (without the LASSO penalty) including only the covariates retained by the LASSO procedure. We examined plots of the Schoenfeld residuals and used *χ* ^2^ tests to assess the proportional hazards assumption (Hosmer et al. 1999); details are provided in Additional File 1.

In order to quantify the contribution of each of the variable domains (demographic, criminal justice, substance use, mental health, physical health, social support, health involvement and socioeconomic factors) to predicting re-incarceration, we compared the discriminative capacity of the final (reduced) multivariate model to the same model without that group of predictors. To quantify the importance of the health-related factors as a whole, we also compared the final multivariate model to a model containing only demographic and criminal justice variables. We compared models by computing the difference in the proportion of explained variation (adjusted for over-optimism induced by the inclusion of additional variables) in the larger model compared to the smaller model, $\varDelta {R}_{adj}^2$, using the str2d function in Stata (Royston 2006). We computed approximate 95% confidence intervals (CIs) for $\varDelta {R}_{adj}^2$ from 1000 bootstrapped samples (Royston and Sauerbrei 2004). Analyses were performed in Stata version 13.0 and R version 3.0.1.

#### Sensitivity analysis

In a sensitivity analysis, we included the re-entry intervention as a variable in our final multivariate Cox model. We checked for an effect of the intervention and any changes in the effect estimates for the other variables included in our model.

## Results

### Descriptive statistics

A total of 1325 participants were enrolled in the study and interviewed at baseline. Our sample was broadly representative of the prisoner population in Queensland, with the exception that women were intentionally over-sampled Australia (Kinner et al. 2013). Records for 1322 participants were successfully linked to QCS re-incarceration data, however three of these participants were not released from prison within the study period and were subsequently excluded. Of the remaining participants (N = 1319), 721 (55%) were re-incarcerated during the study period and 598 (45%) were censored, either because of death (n = 25) or because they had not returned to prison by the study end date (n = 573). This gave a total analysis time of 3301 person-years with a median follow-up of 2.4 years (inter-quartile range: 0.6 to 4.3).

Table 2 shows the distribution of baseline characteristics in the cohort and the percentage of participants who were re-incarcerated within the study period according to these characteristics. Participants were predominantly male (78.8% of the cohort), non-Indigenous (76.4%) and aged between 25 and 39 years (51.2%). Rates of previous contact with the criminal justice system were high (67.1% had a prior incarceration as an adult), as were rates of substance use (55.7% had a history of injecting illicit drugs), mental illness (30.2% were taking CNS medications at baseline), STIs (27.8% reported a historical diagnosis) and chronic illness (58.8% reported historical diagnoses of at least two chronic illnesses). While 20% of the cohort reported low patient activation, less than 3% stated that maintaining either physical or mental health after release was not important. The majority of participants had been exposed to indicators of socioeconomic disadvantage prior to incarceration; 53.1% of the cohort had been unemployed and 46.9% experienced poverty.

**Table 2:** **Percent re-incarcerated within study period by participant baseline characteristics (N=1319)**

**Variable**	**Number (%)**	**Number (%) re-incarcerated**	
		**Exposed**	**Unexposed**
*Demographic*			
Age (years)			
18-24	336 (25.5)	220 (65.5)	-
25-39	682 (51.7)	407 (59.7)	-
40+	301 (22.8)	94 (31.2)	-
Female	279 (21.2)	136 (48.8)	585 (56.3)
Indigenous	335 (25.4)	238 (71.0)	483 (49.1)
*Criminal justice*			
Prior adult incarceration	884 (67.1)	598 (67.7)	121 (27.9)
Juvenile incarceration	364 (27.9)	262 (72.0)	448 (47.6)
Any income from illegal activities†	278 (21.1)	212 (76.3)	508 (48.9)
Longer sentence (≥6 months)	597 (45.5)	328 (54.9)	391 (54.7)
Drug-related sentence (QCS report)	402 (30.7)	276 (68.7)	441 (48.6)
*Substance use*			
High risk drinking†	475 (36.8)	294 (61.9)	402 (49.3)
Risky cannabis use†	611 (46.4)	420 (68.7)	300 (42.4)
Risky amphetamine use†	504 (38.3)	361 (71.6)	358 (44.0)
Risky opioid use†	278 (21.1)	212 (76.3)	506 (48.8)
*Mental health*			
History of self-harm	357 (27.1)	196 (54.9)	525 (54.6)
CNS medications	370 (30.2)	213 (57.6)	448 (52.4)
Screens positive for intellectual disability	398 (30.7)	248 (62.3)	458 (51.0)
High psychological distress	341 (26.0)	187 (54.8)	531 (54.6)
*Physical health*			
Two or more chronic illnesses	776 (58.8)	391 (50.4)	330 (60.8)
Low physical health functioning	326 (25.1)	165 (50.6)	542 (55.7)
Obese	258 (19.7)	104 (40.3)	612 (58.1)
Sedentary	340 (25.9)	178 (52.4)	540 (55.4)
Any STI	367 (27.8)	215 (58.6)	506 (53.2)
*Social support*			
Not married or de-facto	862 (65.4)	487 (56.5)	234 (51.2)
No visits in past four weeks	706 (53.5)	439 (62.2)	282 (46.0)
Low perceived social support	251 (19.1)	155 (61.8)	563 (52.9)
Taken from family as child	262 (19.9)	161 (61.5)	557 (52.9)
*Health involvement*			
Low patient activation	261 (20.0)	170 (65.1)	542 (51.8)
Physical health not important	38 (2.9)	29 (76.3)	690 (54.0)
Mental health not important	33 (2.5)	23 (69.7)	697 (54.3)
*Socioeconomic*			
Less than ten years schooling	569 (43.3)	357 (62.7)	361 (48.4)
Unemployed†	700 (53.1)	436 (62.3)	285 (46.0)
Unstable housing†	271 (20.6)	166 (61.3)	555 (53.0)
Below poverty line†	618 (46.9)	344 (55.7)	376 (53.8)

†prior to index incarceration.

### Cox proportional hazards regression

Table 3 shows the unadjusted hazard ratios (HRs) and adjusted hazard ratios (AHRs) for time to re-incarceration according to baseline characteristics. From among the 32 variables in the full model, four variables (self-rated physical health, importance of mental health post-release, less than ten years of schooling and unemployment) were excluded by the LASSO procedure. Of the 1319 participants for whom re-incarceration data were available, 13% (n = 176) had missing data for one or more of the 28 remaining variables.

**Table 3:** **Hazard ratios from Cox proportional hazards regression on time to re-incarceration**

**Variable**	**Univariate models**	**Full model**	**Reduced model**	$\varDelta {\boldsymbol{R}}_{\boldsymbol{adj}}^2$ **(95%CI)**
	**HR (95%CI)**	**AHR (95%CI)**	**AHR (95%CI)**	
		**(N = 1122)**	**(N = 1143)**	
*Demographic*				0.013 (0.000, 0.035)
Age (years)				
18-24	1.00 (ref.)	1.00 (ref.)	1.00 (ref.)	
25-39	0.86 (0.73, 1.01)	0.77 (0.63, 0.95)	0.78 (0.64, 0.95)	
40+	0.36 (0.28, 0.45)	0.50 (0.37, 0.68)	0.51 (0.38, 0.69)	
Female	0.79 (0.66, 0.96)	0.72 (0.56, 0.93)	0.72 (0.57, 0.92)	
Indigenous	1.75 (1.50, 2.05)	1.22 (0.98, 1.52)	1.22 (0.99, 1.51)	
*Criminal justice*				0.072 (0.044, 0.113)
Prior adult incarceration	3.42 (2.81, 4.17)	2.42 (1.89, 3.10)	2.43 (1.91, 3.11)	
Juvenile incarceration	2.10 (1.80, 2.45)	1.54 (1.27, 1.88)	1.51 (1.25, 1.82)	
Any income from illegal activities†	2.20 (1.87, 2.59)	1.43 (1.11, 1.83)	1.39 (1.10, 1.76)	
Longer sentence (≥6 months)	1.01 (0.88, 1.17)	0.88 (0.74, 1.05)	0.90 (0.75, 1.07)	
Drug-related sentence	1.79 (1.54, 2.08)	1.39 (1.15, 1.67)	1.38 (1.15, 1.66)	
*Substance use*				0.008 (-0.007, 0.034)
High risk drinking†	1.40 (1.20, 1.63)	1.12 (0.93, 1.34)	1.12 (0.94, 1.34)	
Risky cannabis use†	2.03 (1.75, 2.36)	1.28 (1.07, 1.54)	1.27 (1.06, 1.51)	
Risky amphetamine use†	2.17 (1.87, 2.51)	1.24 (1.02, 1.51)	1.20 (0.99, 1.46)	
Risky opioid use†	2.17 (1.85, 2.55)	1.32 (1.08, 1.63)	1.33 (1.08, 1.63)	
*Mental health*				0.023 (0.001, 0.046)
History of self-harm	1.00 (0.85, 1.18)	0.70 (0.57, 0.86)	0.72 (0.59, 0.88)	
CNS medications	1.20 (1.02, 1.41)	1.29 (1.07, 1.57)	1.28 (1.06, 1.54)	
Screens positive for intellectual disability	1.37 (1.17, 1.60)	1.16 (0.96, 1.40)	1.15 (0.96, 1.38)	
High psychological distress	1.01 (0.86, 1.20)	0.89 (0.73, 1.10)	0.92 (0.76, 1.13)	
*Physical health*				0.011 (-0.002, 0.034)
Two or more chronic illnesses	0.75 (0.65, 0.87)	0.87 (0.72, 1.04)	0.85 (0.71, 1.01)	
Low physical health functioning	0.84 (0.71, 1.00)	0.99 (0.80, 1.23)	-	
Obese	0.59 (0.48, 0.72)	0.81 (0.64, 1.02)	0.81 (0.64, 1.02)	
Sedentary	0.86 (0.73, 1.02)	0.82 (0.67, 1.00)	0.82 (0.68, 1.00)	
Any STI	1.18 (1.00, 1.38)	1.20 (0.98, 1.46)	1.18 (0.98, 1.43)	
*Social support*				0.000 (-0.008, 0.016)
Not married or defacto	1.18 (1.01, 1.37)	1.07 (0.88, 1.29)	1.06 (0.88, 1.28)	
No visits in past 4 weeks	1.58 (1.36, 1.83)	1.12 (0.93, 1.34)	1.14 (0.95, 1.37)	
Low perceived social support	1.29 (1.08, 1.55)	1.10 (0.88, 1.37)	1.06 (0.85, 1.31)	
Taken from family as child	1.35 (1.13, 1.61)	0.88 (0.70, 1.09)	0.87 (0.70, 1.08)	
*Health involvement*				0.002 (-0.008, 0.024)
Low patient activation	1.41 (1.19, 1.68)	1.13 (0.92, 1.38)	1.14 (0.94, 1.39)	
Physical health not important	1.83 (1.26, 2.65)	1.61 (0.98, 2.64)	1.52 (0.98, 2.36)	
Mental health not important	1.63 (1.08, 2.48)	0.88 (0.51, 1.52)	-	
*Socioeconomic*				0.003 (-0.005, 0.018)
Less than 10 years schooling	1.52 (1.31, 1.76)	1.00 (0.83, 1.19)	-	
Unstable housing†	1.29 (1.08, 1.53)	1.09 (0.89, 1.34)	1.08 (0.88, 1.31)	
Unemployed†	1.61 (1.39, 1.87)	0.93 (0.77, 1.13)	-	
Below poverty line†	1.05 (0.91, 1.22)	1.29 (1.04, 1.60)	1.24 (1.02, 1.52)	

†prior to index incarceration.

Results from the final (reduced) multivariate model containing 28 variables suggested that older age and female sex were associated with substantial reductions in the risk of return to custody, while Indigenous status was associated with a moderate increase. A history of incarceration as a juvenile or adult, income from illegal activities and having a drug-related sentence were strongly associated with increases in the hazard of re-incarceration, and having a longer sentence was associated with a small reduction. Illicit drug use prior to incarceration exhibited moderate positive associations with risk of return to custody, while risky drinking exhibited a weak positive association. Taking CNS medications and intellectual disability were associated with moderate increases in the hazard of re-incarceration, psychological distress was weakly associated with re-incarceration and a history of self-harm was associated with a decreased hazard. Among indicators of physical health, chronic illness, obesity and sedentary behaviour were associated with moderate reductions in the hazard of return to custody, while ever having been diagnosed with an STI was associated with a moderate increase. Social support factors showed only weak associations with return to custody. Low patient activation exhibited a weak positive association, while reporting that maintaining physical health after release was not important was strongly associated with increased risk of return to custody. Unstable housing was weakly associated with return to custody, and being below the poverty line prior to incarceration showed a moderate positive association.

**Figure 1:**
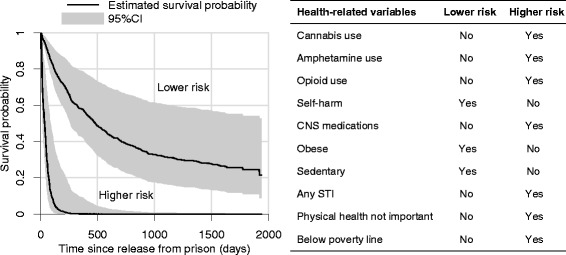
**Estimated survival curves after multivariate Cox proportional hazards regression.** The plot shows the probability of re-incarceration against time since release from prison for two hypothetical individuals, estimated using the reduced multivariate Cox model presented in Table 3. The individuals represented are exposed to the same demographic and criminal justice risk factors for re-incarceration (<25 years of age, male, Indigenous, prior adult and juvenile incarceration, income from illegal activities, sentence <6 months, drug-related sentence). The differences in their survival curves are entirely explained by differences in the health-related factors with the ten largest effect sizes, as described in the table at right. Both profiles are not exposed to the remaining health-related variables included in the reduced model.

Figure 1 illustrates the combined effect size of the key health-related predictors. The plot shows the estimated probability of avoiding re-incarceration (survival probability) against time since release for two types of participants with differing baseline characteristics. These two risk profiles are the same with respect to demographic and criminal justice risk factors for re-incarceration. The ‘high-risk’ profile is exposed to the health-related risk factors that had the ten largest effect sizes (absolute value of the log hazard ratio) according to the reduced model of Table 3. The ‘low-risk’ profile is exposed to none of these, and both profiles are not exposed to the remaining health-related factors. The graph illustrates the stark difference in risk of re-incarceration between the two profiles. For the low-risk profile, the risk of returning to prison within three months was estimated to be 13% (95%CI 6, 19); for the high-risk profile, the estimated risk was 84% (95%CI 48, 95), a more than six-fold increase.

Tests of the proportional hazards assumption via the Schoenfeld residuals for the reduced model suggested that this assumption was violated for the variables Indigenous status and sedentary. We therefore ran a new model including a time-varying HR for each of these variables. Our qualitative interpretation of the results from this model did not change substantively from the time-constant model; full results from the time-varying model are shown in Additional File 1.

#### Predictive capacity of the models

**Figure 2:**
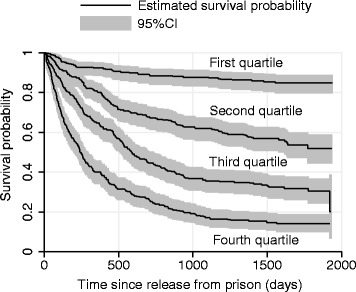
**Kaplan-Meier survival curves by quartile of linear predictor from reduced multivariate Cox model.** To produce this plot, we calculated the linear predictor from the reduced multivariate Cox model presented in Table 3 for the N = 1143 individuals for whom all relevant variables were available. We then categorised participants based on quartiles of this linear predictor, and produced Kaplan-Meier curves for each group.

Figure 2 demonstrates the discriminative capacity of the final (reduced) multivariate model graphically by presenting Kaplan-Meier curves for groups of participants categorised according to quartiles of the linear predictor (sum of the estimated log hazard ratios multiplied by the covariate values for each participant). We compared this model to a model including only demographic and criminal justice predictors of recidivism. The adjusted proportion of explained variation was $\varDelta {R}_{adj}^2$ = 0.239 for the demographic and criminal justice model and $\varDelta {R}_{adj}^2$ = 0.290 for the model that also included health-related variables, an increase of $\varDelta {R}_{adj}^2$ = 0.051 (95%CI 0.031, 0.107). As shown in Table 3, variables in the mental health ($\varDelta {R}_{adj}^2$ = 0.023; 95%CI 0.001, 0.046), physical health ($\varDelta {R}_{adj}^2$ = 0.011; 95%CI -0.002, 0.034) and substance use ($\varDelta {R}_{adj}^2$ = 0.008; 95%CI −0.007, 0.034) domains were the biggest contributors to this improvement in model discrimination. Overall, variables in the criminal justice domain yielded the largest increase in ($\varDelta {R}_{adj}^2$= 0.072; 95%CI 0.044, 0.113).

### Sensitivity analysis

In a sensitivity analyses, we included receipt of the re-entry intervention in our final multivariate model. We found no evidence for an effect of the intervention on time to re-incarceration (HR 1.05; 95%CI 0.89, 1.24) and the effect estimates for the other variables were virtually unchanged.

## Discussion

This study has identified a number of novel, health-related factors that predict re-incarceration in a large cohort of ex-prisoners. Many of the factors identified are easily measureable prior to release from prison, and could inform improvements to transitional planning without the need for additional, in-depth assessment. Most of these predictors are also modifiable and could potentially serve as targets for re-entry interventions. However, this study was exploratory and further investigation into the causal nature of the observed associations is recommended.

In our study, participants who reported risky use of cannabis, amphetamines or opioids prior to incarceration or who had committed a drug-related crime had an increased risk of re-incarceration. These substance use factors frequently co-occurred in our sample − 43% reported at least two of these risk factors − and their combined effect was substantial. Taking CNS medications, a proxy for diagnosed mental disorder, was associated with a similar increase in re-incarceration rate. These findings confirm and build on previous research identifying a positive association between substance use, mental disorder and recidivism (Dowden and Brown 2002; Baillargeon et al. 2010). The observed protective effect of a history of self-harm (including attempted suicide) is novel, unexpected and at odds with our other findings; further investigation is required.

Intellectual disability was also associated with a marginally increased risk of return to custody in this study. While there is evidence that people with intellectual disabilities are over-represented in prisons in Western countries (Lindsay 2002), it has been suggested that the proportion of prisoners with an intellectual disability may be inflated by the increased risk of re-offending in this group (Hayes 1996). Our findings are consistent with this interpretation.

In our sample, sedentary behaviour, obesity and chronic disease were associated with a reduced rate of re-incarceration, and these effects were not accounted for by gender or age. One possible explanation is a version of the healthy worker effect: those who are physically healthy may be more able to commit certain crimes and might therefore be at higher risk of re-incarceration. This effect has previously been proposed as an explanation for lower rates of natural deaths during incarceration (Fazel and Benning 2006). Conversely, a history of STI was associated with a marginal increase in risk of return to custody. Most STIs are treatable and not physically debilitating, such that the ‘healthy offender’ effect is unlikely to apply. Instead, a possible explanation for the observed positive association with re-offending is that STIs are a marker for risky sexual behaviour, which may in turn be correlated with a number of other risk factors for recidivism. Previous research has revealed high rates of both STIs (comparable to the rate of 28% found in our cohort) and sexual risk behaviour among Australian prisoners (Butler et al. 2013).

Participants who reported that maintaining their health post-release was not important had a 50% greater hazard of re-incarceration. This observation may indicate that those who are committed to their own health and wellbeing are less likely to return to custody. However, the vast majority of participants (97%) reported that post-release health was important to them, suggesting that the remaining 3% represented an extreme subset of the cohort.

Consistent with previous research in Australia (Broadhurst et al. 1988), Indigenous participants had an increased hazard of re-incarceration. Indigenous people are over-represented in Australian prisons by an age-adjusted factor of 15 (ABS 2014) and evidence suggests that social disadvantage, substance misuse and poor health are important drivers of this disparity (Krieg 2006). In our study, the association between Indigenous status and re-incarceration was attenuated when these factors were taken into account, but remained significant. One possible explanation for these findings relates to discrimination: there is good evidence that, independent of measured risk factors, Indigenous people have been subjected to systematic bias by the police and courts in Australia (Weatherburn et al. 2003). To the extent that this is the case, this finding highlights the importance of implementing structural as well as individual-level reforms to reduce re-incarceration in ex-prisoners.

Finally, being below the poverty line prior to index incarceration was identified as a risk factor for return to custody, consistent with existing evidence demonstrating a link between socioeconomic disadvantage and incarceration (Travis et al. 2014). The period of incarceration provides a potential opportunity to intervene and break the cycle of disadvantage and imprisonment, including through education and vocational training, and training in basic financial skills (Vacca 2004; Visher and Travis 2003). However, education and unemployment prior to incarceration were not associated with return to custody in our cohort, consistent with at least one previous study (Fu et al. 2013).

Overall, the combined predictive capacity of the health-related predictors identified in our study was substantial. Inclusion of these predictors in a prognostic model for recidivism considerably improved discriminative capacity. Moreover, compared with exposure to criminal justice and demographic risk factors only, exposure to ten key health-related risk factors produced an estimated six-fold increase in risk of re-incarceration within three months of release. This is a large effect given the burden to both society and individuals of repeated incarceration. These findings highlight the important intersection of criminal justice and public health priorities: targeting modifiable, health-related factors during and after the transition from prison to community may have the potential to simultaneously improve both health and public safety.

Variables related to mental health provided the greatest contribution overall to this increase in ability to predict re-incarceration. Extensive previous research has established that mentally disordered offenders are at increased risk of recidivism (Fazel and Yu 2011; Baillargeon et al. 2010; Yu et al. 2012). While our effect estimates indicate the presence of associations between the other health domains studied and re-incarceration, when a bootstrapping technique was used to account for over-fitting, the contribution of these domains to predicting return to custody was less clear. In addition, for some health-related variables, notably history of self-harm, the direction of observed association was not as expected. Further research is needed to establish the reproducibility of our findings and to understand the causal pathways (if any) linking these health-related factors to recidivism.

### Limitations

This study was limited by several factors. First, we relied on self-report measures for most baseline variables, which may be subject to recall or social desirability bias. This may have resulted in under-reporting of stigmatised behaviours. However, for many stigmatised behaviours such as pre-prison illicit drug use, self-report is the only feasible approach and can be reliable (Darke 1998).

Second, 13% of participants were missing data for one or more baseline variables included in our final multivariate model, and were hence excluded. Missingness was due largely to the prescription CNS medication variable, which was omitted for the 7% of participants who did not give consent for the researchers to access their prison health records. If missingness for a given predictor was associated with both the predictor and the outcome (re-incarceration), this could bias our results. However, since missingness for any variable was at most 7%, the effect of any such bias is likely to be small.

Third, we did not have access to records of re-incarcerations outside the state of Queensland, and as such may have under-ascertained the outcome. Furthermore, death index data were only available up to July 2013 and thus did not cover the full follow-up period considered in this study, leading to possible under-ascertainment of deaths (and therefore over-estimation of time at risk) in our cohort. However, in both cases we expect relatively few such events and there is no a priori reason to suspect that they would bias the measures of association presented here.

Finally, we included parole violations as re-incarceration events in the present study, which may have very different causes to re-incarceration for new crimes. While all-cause re-incarceration is a valid and useful outcome measure, studying parole violations and new offences separately is necessary in order to understand pathways to re-incarceration more fully. Future research should account for this where possible.

## Conclusions

In a large cohort of Australian prisoners, health-related characteristics including mental health, physical health and substance use were important predictors of re-incarceration in addition to well-documented demographic and criminal justice risk factors. The combined contribution of these health-related variables to predicting return to custody was substantial. However, the direction of the associations between health measures and re-incarceration was not as expected in all cases. More targeted research is required to establish the reproducibility of our findings and to understand the possible causal pathways linking health-related outcomes in prisoners and ex-prisoners to re-incarceration.

## Additional file

Additional File 1:
**Tests of the proportional hazards assumption.** The Additional File describes our approach to testing the proportional hazards assumption for the reduced multivariate Cox model presented in Table 3. The File presents results from these tests and shows hazard ratios from a time-varying Cox model which resolves violations of the proportional hazards assumption in the reduced model.

## Abbreviations

(A)HR: (Adjusted) hazard ratio
CI: Confidence interval
CNS: Central nervous system
HIV: Human immunodeficiency virus
QCS: Queensland Correctional Services
RCT: Randomised controlled trial
STI: Sexually transmitted infection
