# Supplementary material for: Health-related factors predict return to custody in a large cohort of ex-prisoners: new approaches to predicting re-incarceration
Source: Health Justice. 2015 May 20;3:10. doi: 10.1186/s40352-015-0022-6 (PMC5151521; doi:10.1186/s40352-015-0022-6)
Supplement: Additional File 1: — Tests of the proportional hazards assumption. The Additional File describes our approach to testing the proportional hazards assumption for the reduced multivariate Cox model presented in Table 3. The File presents results from these tests and shows hazard ratios from a time-varying Cox model which resolves violations of the proportional hazards assumption in the reduced model. [file 40352_2015_22_MOESM1_ESM.pdf]

Health-related factors predict return to custody in a large cohort of ex-prisoners: new approaches to predicting re-incarceration

Additional File 1: Tests of the proportional hazards assumption

Emma G Thomas, Matthew J Spittal, Faye S Taxman, Stuart A Kinner

For the final reduced multivariate Cox proportional hazards model presented in Table 3 of the main text, we assessed the proportional hazards assumption via the Schoenfeld residuals. We examined plots of the residuals for each variable against time to check for a non-zero slope, which indicates that the proportional hazards assumption is violated (Hosmer et al. 1999). We then performed statistical tests of this assumption via a generalised linear regression of the residuals against time.

A global test of proportional hazards for the reduced model provided some evidence that this assumption was violated ($\chi^2(29)$ = 43.04, *p* = 0.0451), and tests for individual variables (Table S1) indicated that it was most strongly violated for Indigenous status ($\chi^2(1)$ = 9.96, *p* = 0.0016) and being sedentary ($\chi^2(1)$ = 9.07, *p* = 0.0026). A plot of the Schoenfeld residuals for Indigenous status vs. time (Figure S1) and sedentary vs. time (Figure S2) indicated that the HRs for both of these variables were different after approximately 500 days following release. We therefore ran a new model including a time-varying HR (split at 500 days after release) for Indigenous status and sedentary. A likelihood ratio test comparing this model to the time-constant version provided strong evidence against the null hypothesis of time constant HRs for these variables (*p* = 0.0013). Repeating the global test of proportional hazards for the new model provided no evidence against this assumption ($\chi^2(31)$ = 28.57, *p* = 0.5916).

Adjusted hazard ratios (AHRs) from the time-varying model are shown in Table S2. The results suggested that being Indigenous was not associated with return to custody up to 500 days after release (AHR 1.02; 95%CI 0.79-1.30), but was associated with an 83% increase in

the hazard of re-incarceration thereafter (AHR 1.83; 95%CI 1.31, 2.55). Conversely, being sedentary was associated with a 30% reduction in the hazard of re-incarceration up to 500 days after release (AHR 0.72; 95%CI 0.57-0.91), but was not associated with return to custody thereafter (AHR 1.06; 95%CI 0.77, 1.47). The estimated HRs for the other variables were very similar to the time-constant model

References

Hosmer, D., Lemeshow, S., & May, S. (1999). *Applied survival analysis: regression modeling of time to event data*. New York: John Wiley&Sons.

Table S1: Tests of the proportional hazards assumption for the reduced model presented in Table 3 of the main text.

**Variable**	χ^2	**Df**	***P*-value**
Age (years)			
25-39	0.29	1	0.5914
40+	0.02	1	0.8745
Female	0.21	1	0.648
Indigenous	9.96	1	0.0016
Prior adult incarceration	0.61	1	0.4365
Juvenile incarceration	1.67	1	0.1962
Any income from illegal activities†	2.15	1	0.1424
Longer sentence (≥6 months)	0.52	1	0.4691
Drug-related sentence	0.58	1	0.4465
High risk drinking†	0.23	1	0.6345
Risky cannabis use†	4.19	1	0.0407
Risky amphetamine use†	0.07	1	0.7862
Risky opioid use†	1.18	1	0.2777
History of self-harm	0.65	1	0.4218
CNS medications	3.19	1	0.0743
Screens positive for intellectual disability	0.01	1	0.9074
High psychological distress	0.81	1	0.3672
Two or more chronic illnesses	0.12	1	0.7306
Obese	1.11	1	0.2915
Sedentary	9.07	1	0.0026
Any STI	0.22		0.6425
Not married or defacto	0.01	1	0.9059
No visits in past 4 weeks	0.24	1	0.6235
Low perceived social support	0.60	1	0.4369
Taken from family as child	3.63	1	0.0568
Low patient activation	0.39	1	0.53
Physical health not important	0.34	1	0.5577
Unstable housing†	0.01	1	0.9136
Below poverty line†	3.40	1	0.0651
Global test	43.04	29	0.0451

†prior to incarceration

Caption: The table presents chi-squared tests of non-zero coefficients from generalised linear regressions of the Schoenfeld residuals for each variable against time. The global result tests the slopes for all variables simultaneously. Results were generated using the *estat phtest* command in Stata.

Table S2: Results from the reduced multivariate model with time-constant and time-varying HRs (N=1153)

Variable	Time-constant model AHR (95%CI)	Time-varying model AHR (95%CI)
Age (years)		
18-24	1.00 (ref.)	1.00 (ref.)
25-39	0.78 (0.64, 0.95)	0.78 (0.64, 0.95)
40+	0.51 (0.38, 0.69)	0.51 (0.38, 0.69)
Female	0.72 (0.57, 0.92)	0.72 (0.56, 0.91)
Indigenous (≤500 days after release)	1.22 (0.99, 1.51)	1.02 (0.79, 1.30)
Indigenous (>500 days after release)	As above	1.83 (1.31, 2.55)
Prior adult incarceration	2.43 (1.91, 3.11)	2.40 (1.88, 3.06)
Juvenile incarceration	1.51 (1.25, 1.82)	1.52 (1.26, 1.84)
Any income from illegal activities†	1.39 (1.10, 1.76)	1.39 (1.10, 1.76)
Longer sentence (≥6 months)	0.90 (0.75, 1.07)	0.90 (0.75, 1.07)
Drug-related sentence	1.38 (1.15, 1.66)	1.39 (1.15, 1.67)
High risk drinking†	1.12 (0.94, 1.34)	1.12 (0.94, 1.34)
Risky cannabis use†	1.27 (1.06, 1.51)	1.27 (1.06, 1.52)
Risky amphetamine use†	1.20 (0.99, 1.46)	1.20 (0.99, 1.45)
Risky opioid use†	1.33 (1.08, 1.63)	1.32 (1.08, 1.62)
History of self-harm	0.72 (0.59, 0.88)	0.72 (0.59, 0.88)
CNS medications	1.28 (1.06, 1.54)	1.27 (1.05, 1.54)
Screens positive for intellectual disability	1.15 (0.96, 1.38)	1.15 (0.96, 1.37)
High psychological distress	0.92 (0.76, 1.13)	0.92 (0.75, 1.12)
Two or more chronic illnesses	0.85 (0.71, 1.01)	0.85 (0.71, 1.02)
Obese	0.81 (0.64, 1.02)	0.80 (0.63, 1.01)
Sedentary (≤ 500 days after release)	0.82 (0.68, 1.00)	0.72 (0.57, 0.91)
Sedentary (>500 days after release)	As above	1.06 (0.77, 1.47)
Any STI	1.18 (0.98, 1.43)	1.19 (0.98, 1.44)
Not married or defacto	1.06 (0.88, 1.28)	1.06 (0.88, 1.27)
No visits in past 4 weeks	1.14 (0.95, 1.37)	1.15 (0.96, 1.37)
Low perceived social support	1.06 (0.85, 1.31)	1.05 (0.85, 1.31)
Taken from family as child	0.87 (0.70, 1.08)	0.87 (0.71, 1.08)
Low patient activation	1.14 (0.94, 1.39)	1.14 (0.94, 1.39)
Physical health not important	1.52 (0.98, 2.36)	1.57 (1.01, 2.44)
Unstable housing†	1.08 (0.88, 1.31)	1.09 (0.89, 1.33)
Below poverty line†	1.24 (1.02, 1.52)	1.25 (1.02, 1.52)

†prior to index incarceration

Caption: The table presents the estimated hazard ratios from a time-varying model in which two HRs are fit for each of the variables Indigenous status and sedentary: one for ≤ 500 days after release, and one for > 500 days after release. These HRs are highlighted in light grey. For comparison, the results from the model without the time-varying HR are shown (original model; also shown in Table 3 of the main text).

Figure S1: Schoenfeld residuals for the variable Indigenous status from the reduced multivariate model, against time.

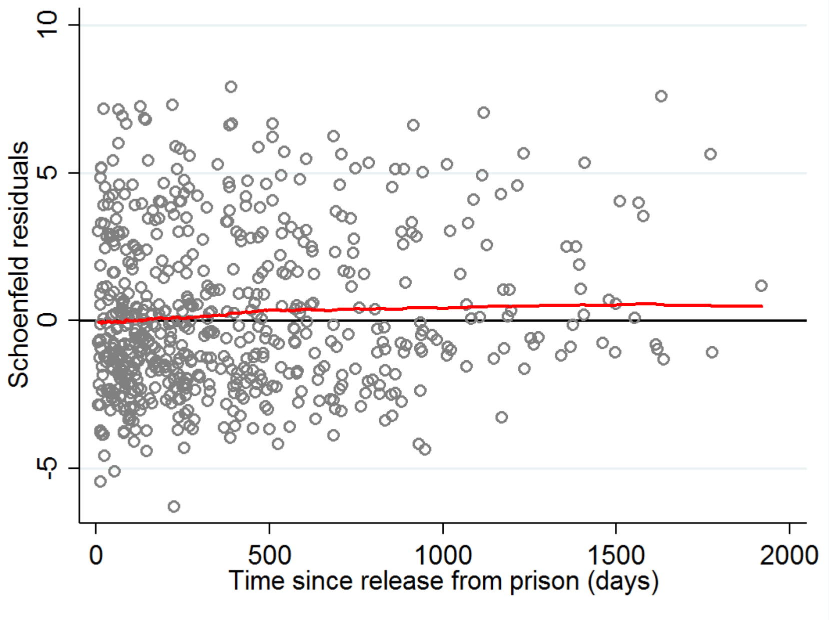

Caption: The circles are the Schoenfeld residuals for Indigenous status, and the line represents a lowess-smoothed line of best fit.

Figure S2: Schoenfeld residuals for the variable sedentary from the reduced multivariate model, against time.

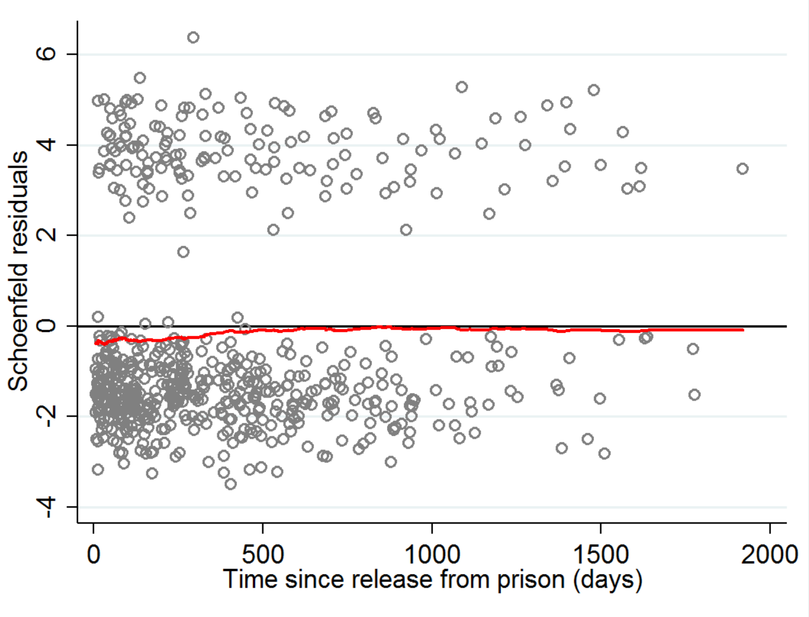

Caption: The grey circles are the Schoenfeld residuals for Indigenous status, and the line represents a lowess-smoothed line of best fit.
